# Identification of Reference Genes for Precise Expression Analysis during Germination in *Chenopodium quinoa* Seeds under Salt Stress

**DOI:** 10.3390/ijms242115878

**Published:** 2023-11-01

**Authors:** Estefanía Contreras, Lucía Martín-Fernández, Arafet Manaa, Jesús Vicente-Carbajosa, Raquel Iglesias-Fernández

**Affiliations:** 1 Centro de Biotecnología y Genómica de Plantas-Severo Ochoa (CBGP, UPM-INIA/CSIC), Universidad Politécnica de Madrid (UPM)—Instituto Nacional de Investigación y Tecnología Agraria y Alimentaria (INIA/CSIC), Campus de Montegancedo, Pozuelo de Alarcón, 28223 Madrid, Spain; estefania.contreras@upm.es (E.C.); jesus.vicente@upm.es (J.V.-C.); 2 Laboratory of Extremophile Plants, Centre of Biotechnology de Borj Cedria, B.P. 901, Hammam-Lif 2050, Tunisia; arafet.manaa@cbbc.rnrt.tn; 3 Departamento de Biotecnología-Biología Vegetal, Escuela Técnica Superior de Ingeniería Agronómica, Alimentaria y de Biosistemas (UPM), 28040 Madrid, Spain

**Keywords:** *Chenopodium quinoa*, germination *sensu stricto*, reference gene, quantitative PCR, salt stress, seed

## Abstract

*Chenopodium quinoa* Willd. (quinoa), a member of the Amaranthaceae family, is an allotetraploid annual plant, endemic to South America. The plant of *C. quinoa* presents significant ecological plasticity with exceptional adaptability to several environmental stresses, including salinity. The resilience of quinoa to several abiotic stresses, as well as its nutritional attributes, have led to significant shifts in quinoa cultivation worldwide over the past century. This work first defines germination *sensu stricto* in quinoa where the breakage of the pericarp and the testa is followed by endosperm rupture (ER). Transcriptomic changes in early seed germination stages lead to unstable expression levels in commonly used reference genes that are typically stable in vegetative tissues. Noteworthy, no suitable reference genes have been previously identified specifically for quinoa seed germination under salt stress conditions. This work aims to identify these genes as a prerequisite step for normalizing qPCR data. To this end, germinating seeds from UDEC2 and UDEC4 accessions, with different tolerance to salt, have been analyzed under conditions of absence (0 mM NaCl) and in the presence (250 mM NaCl) of sodium chloride. Based on the relevant literature, six candidate reference genes, *Glyceraldehyde-3-phosphate dehydrogenase* (*GAPDH*), *Monensin sensitivity1* (*MON1*), *Polypyrimidine tract-binding protein* (*PTB*), *Actin-7* (*ACT7*), *Ubiquitin-conjugating enzyme* (*UBC*), and *18S ribosomal RNA* (*18S*), were selected and assessed for stability using the *RefFinder* Tool encompassing the statistical algorithms *geNorm*, *NormFinder*, *BestKeeper*, and *ΔCt* in the evaluation. The data presented support the suitability of *CqACT7* and *CqUBC* as reference genes for normalizing gene expression during seed germination under salinity stress. These recommended reference genes can be valuable tools for consistent qPCR studies on quinoa seeds.

## 1. Introduction

*Chenopodium quinoa* Willd. (quinoa), a member of the Amaranthaceae family (dicotyledonous), is an allotetraploid annual plant [1], endemic to South America, that was cultivated by indigenous communities in the Andean Region for millennia. Quinoa is commonly recognized as a pseudo-cereal and eventually as a pseudo-oilseed due to its composition in starch, gluten-free protein, and fatty acids. The grain boasts superior protein quality compared to wheat, barley, and soybean, ensuring a comprehensive and rich profile of essential amino acids. Nonetheless, genotype has been found to affect seed composition, limiting certain essential amino acids. Additionally, its nutritional value is further enhanced by a well-balanced combination of fatty acids, minerals, vitamins, and antioxidants [2,3,4].

Abiotic stresses including drought, high temperature, salinity, or ultraviolet radiation can induce plant membrane injury, protein denaturation, and reactive oxygen species (ROS) formation. These stresses affect plant growth and health, contributing to yield loss [5]. Salinity is a critical environmental issue that affects a substantial portion of the Earth’s terrestrial surface. It is estimated that roughly 7% of the world’s land area grapples with salinity-related issues. The primary contributors to salinity include highly soluble compounds such as sodium chloride (NaCl), calcium, and magnesium chlorides. Soil salinization and sodification are two prevalent processes associated with salinity that are particularly pronounced in arid regions [6]. The plant of *C. quinoa* presents significant ecological plasticity, thriving in diverse geographical areas spanning from sea level to approx. 4000 m, and with exceptional adaptability to environmental stresses such as drought, cold, and salinity [7,8]. Indeed, it is considered a halophytic plant, and the specific tolerance mechanisms are still unknown. However, the epidermal bladder cells (EBCs), densely covering the shoot surface of the plant, mediate salt tolerance by serving as salt dumps [9,10]. Additionally, two studies have identified transmembrane domain-enriched and chitinase-like proteins associated with salt tolerance that eventually can be used as biomarkers of drought [11,12]. The resilience of quinoa to diverse abiotic stresses, coupled with its exceptional nutritional attributes, has triggered substantial shifts in global quinoa cultivation over the past century, experiencing an expansion of over 120 countries. This transformation has positioned quinoa as a promising resilient crop for coping with climate challenges like in regions affected by drought and salinity [13]. However, it has been described that several accessions of quinoa seeds encounter vulnerabilities throughout this phase when confronting several biotic and abiotic stresses [8]. 

The seed stage is an essential phase in the plant life cycle that plays an outstanding role in the adaptation to diverse and challenging environments across vast distances [14]. Quinoa grains (achenes) are indehiscent one-seeded fruits where the thin pericarp encloses the seed (botanically defined). Quinoa grains (hereafter seeds) comprise three distinct components: the embryo-surrounding tissues (pericarp, testa, and the remnant endosperm), embryo, and perisperm. The pericarp contains saponins that contribute to the bitter taste experienced during consumption [15]. The embryo constitutes approximately 30% of the seed volume and consists of two cotyledons and the radicle, encircling the perisperm. The perisperm functions as the primary storage tissue, replacing the conventional endosperm, and it contains starch granules, accounting for nearly 60% of the seed content [8]. Although germination is a pivotal event in the life cycle of plants and dictates the timing and conditions for radicle emergence [16], the germination process in *Chenopodium quinoa* seeds has not been fine-tuned assessed, compared with other plant model species such as *Arabidopsis thaliana* or *Brachypodium distachyon* [17,18].

Quantitative PCR (qPCR) is one of the molecular biology techniques most frequently used for the quantification of gene expression due to its remarkable sensitivity and reproducibility. However, the accuracy of qPCR results hinges on the essential step of normalization, which entails selecting reference genes with consistent expression levels across different experimental conditions. Failure to carefully choose these reference genes can result in significant fluctuations in the measured expression levels of target genes [19]. Although researchers often utilize endogenous controls known as housekeeping genes (actin, tubulin, polyubiquitin, and elongation factor 1-α), their stability under all experimental conditions cannot be assumed [20]. Consequently, it is crucial to systematically validate the suitability of reference genes to ensure accurate data normalization. Significant transcriptomic changes during the early stages of germination modify the expression of commonly used reference genes used in vegetative tissues since they display unstable expression levels in seeds [21,22,23].

The validation of traditional and new reference genes for seed germination under control and stress conditions is needed, even more in quinoa, where this process is poorly studied and commonly occurs under harsh environmental conditions. Prior research has identified isocitrate dehydrogenase enzyme (*IDH-A*) and polypyrimidine tract-binding protein (*PTB*) as suitable reference genes for normalizing diurnal expression data in vegetative tissues of Titicaca and Chen-109 quinoa accessions [24]. This study aims to identify suitable reference genes for normalizing germination expression data in *C. quinoa* in control conditions and in the presence of 250 mM NaCl using UDEC2 and UDEC4 accessions (originated from Chile; ochre and yellow color grain, respectively) [25]. Six candidate reference genes (*Glyceraldehyde-3-phosphate dehydrogenase* (*GAPDH*), *Monensin sensitivity 1* (*MON1*), *Polypyrimidine tract-binding protein* (*PTB*), *Actin-7* (*ACT7*), *Ubiquitin-conjugating enzyme* (*UBC*), and *18S ribosomal RNA* (*18S*)) were selected and assessed for stability using the RefFinder Tool that compares four statistical algorithms (*geNorm*, *NormFinder*, *BestKeeper*, and *ΔCt*). The selected reference genes were further validated by normalizing the expression data of quinoa inducible genes *CqMAN7* and *CqABI5* orthologs to those in *A. thaliana* encoding *endo-beta-mannanase 7* (*MAN7*) and *ABA insensitive 5* (*ABI5*) [17,26], expressed in germinating seeds and vegetative tissues under abiotic stress. 

The study presented offers substantial data obtained from germination assays and gene expression analysis, elucidating the impact of varying salt stress levels on the germination process and gene expression patterns in *Chenopodium quinoa* seeds. The utilization of multiple replicates and comprehensive techniques, including qPCR, fortifies the robustness of the dataset. The information is thoughtfully organized, facilitating the drawing of meaningful conclusions, and enabling potential comparisons with other research endeavors. Furthermore, this work provides a thorough account of the techniques employed, encompassing plant material and growth conditions, light microscopy protocols, germination assays, total RNA isolation, primer design, quantitative PCR (qPCR), and gene stability analysis. These methods collectively encompass a diverse array of approaches utilized in the examination of *C. quinoa* seeds under various conditions, such as germination and exposure to salt stress.

## 2. Results and Discussion

### 2.1. Seed Germination of Chenopodium quinoa UDEC2 and UDEC4 Accessions in Response to Salt Stress

#### 2.1.1. Anatomical and Histological Structure of Quinoa Imbibed Seeds

**Figure 1:**
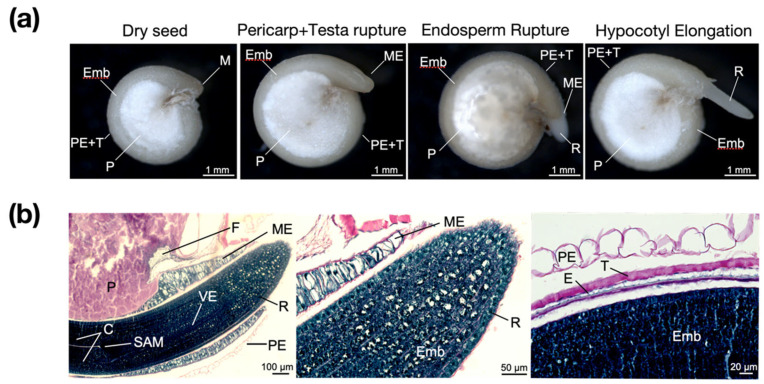
*Chenopodium quinoa* seed germination. (**a**) Different phases of *C. quinoa* (UDEC4) germination *sensu stricto*: Dry seed, Pericarp and Testa rupture Endosperm Rupture, and Hypocotyle Elongation. (**b**) Bright field microscopy of longitudinal sections *of Chenopodium quinoa* germinated seeds stained with PAS-NBB, showing the radicle protrusion (left and center images) and a close-up of the seed coat structure (right image). C, cotyledons; E, endosperm; Emb, embryo; F, funiculus; M, micropyle; ME, micropylar endosperm; P, perisperm; PE, pericarp; R, radicle; SAM, shoot apical meristem; T, testa; VE, vascular elements.

The mature seeds of the *Chenopodium quinoa* are perispermic. Hence, this tissue replaces, at the center of the seed, the endosperm as the main storage tissue [27]. At the macroscopic level, the quinoa seed presents three differentiated structures: the embryo-covering layers (pericarp, testa, and remnant endosperm), the embryo, and the perisperm (Figure 1a). This structure is typical for seeds of the Amaranthaceae, and it can be observed in other members of the family such as *Amaranthus* spp. and *Beta vulgaris* (wild sea beet) [28,29].

Longitudinal sections of imbibed quinoa seeds (18 h of imbibition, hoi) have been stained with Periodic Acid-Schiff-Naphthol Blue Black (PAS-NBB; Figure 1b). PAS reaction stains polysaccharides in pink (starch and cellulose) and NBB is specific for staining proteins in dark blue [18]. The embryo is surrounded by the pericarp, the testa (comprised of inner and outer integuments), and the endosperm. While the micropylar endosperm consists of two cell layers enclosing the radicle tip, the periphery endosperm is a monolayer closely attached to the seed coat (Figure 1b). The embryo-surrounding tissues (pericarp, testa, and remnant endosperm) constitute a fragile and thin structure, except for the micropylar region where the endosperm consists of at least a two-cell layer (Figure 1b), similar to *Beta vulgaris* seeds [29]. While the perisperm cells store mainly starch, the embryo and the endosperm cells accumulate protein and lipids [28]. When the radicle emerges, the protein bodies start to degrade as indicated by the empty cells at the radicle tip (Figure 1b). A similar mechanism has been described in other species like *Arabidopsis thaliana* and *Brachypodium distachyon* where protein bodies are fully degraded at the radicle and the root tips, respectively [18,30]. In *Arabidopsis thaliana*, the hypocotyl–radicle transition zone has been reported as a region of intense activity of cell expansion upon seed germination. In this zone, protein storage vacuoles are rapidly replaced by a central lytic vacuole enabling the elongation of embryonic cells [31]. 

#### 2.1.2. Germination *Sensu Stricto* in *Chenopodium quinoa* Seeds under Salt Stress

Germination *sensu stricto* in quinoa occurs when the micropylar endosperm breaks (Figure 1) following the pericarp and testa breakage (two-step germination). Thus, endosperm rupture (ER) has been established as the criterion for scoring seed germination (Figure 1a,b). The same criterion has been used in several species belonging to Brassicaceae (*Arabidopsis thaliana*, *Brassica rapa*, *Lepidium sativum*, and *Sisymbrium officinale*) [17,32,33] and Solanaceae (*Solanum lycopersicum*) [34].

In *Beta vulgaris* and *Chenopodium album* seeds (Amarantaceae), germination *sensu stricto* proceeds in a similar manner to *C. quinoa*, where testa breakage occurs before the ER [29,35]. In *Poaceae* species (*Avena fatua*, *Brachypodium distachyon*, and *Hordeum vulgare*), the coleorhiza, a tissue analogous to the endosperm, is broken after grain-covering layers do, and coleorhiza rupture has been established as the criterion for scoring seed germination. Both the endosperm in Brassicaceae and the coleorhiza in Poaceae have been described as tissues regulating seed dormancy and germination, mainly by controlling abscisic acid (ABA) responses [36,37]. Whether the micropylar endosperm in quinoa has a similar function to that in other dicotyledonous seeds is a question that requires clarification. Therefore, understanding the precise germination process in quinoa seeds is essential not only to establish a unified accepted criterion, but also to unravel the cellular, molecular, and physiological factors governing a poorly understood process in this orphan crop.

Management of salt-affected areas demands an integrated approach that includes the selection of salt-tolerant crops and plants including halophytes. Soil salinity and sodification are characteristic of dryland regions, where seed germination and seedling establishment are directly affected [6,38]. In this work, germination assays have been conducted on two quinoa (UDEC2 and UDEC4) accessions, characterized by ochre and yellow grains, respectively (see Supplementary Figure S1). The selection of UDEC2 and UDEC4 accessions was based on their NaCl tolerance degree during seed germination, scored from a collection of 214 quinoa genotypes (data not shown; kindly donated by *Quinoa4Med Consortium*; https://quinoa4med.uni-hohenheim.en/; accessed on 28 July 2023). The UDEC4 accession presents high seed yield, harvest index, seed perimeter, and seed area, similar to those of the commercial variety Regalona-Baer [25]. 

Initially, two-year-old seeds were used in germination assays under control (0 mM NaCl) and two salt stress conditions (150 mM and 250 mM NaCl; Supplementary Figure S2). The 150 to 250 mM NaCl range was chosen after a literature search, disclosing a delay in germination but a minimal impact on the maximum germination rate, which facilitates the analysis of the NaCl treatment in quinoa seeds [39,40,41,42,43]. Furthermore, salinity concentrations are deemed elevated at 150 mM NaCl and extreme at 250 mM NaCl when present in soils [44]. 

Germination analyses were focused on comparisons of the t_50_ value (time to get 50% of germination) and the maximum germination percentage (% MG) of two-year-old UDEC2 and UDEC4 seeds. The results in Supplementary Figure S2 show that UDEC2 and UDEC4 seeds exhibit significantly slower germination at 250 mM NaCl (t_50_ = 85.5 ± 5.0 h and t_50_ = 57.0 ± 3.9 h, respectively) compared to 150 mM NaCl (t_50_ = 38.1 ± 5.6 h and t_50_ = 43.6 ± 0.5 h, respectively) and control conditions (0 mM NaCl; t_50_ = 21.1 ± 0.2 h and t_50_ = 20.7 ± 0.8 h, respectively). In addition, while UDEC4 reaches 100% germination in all conditions tested, UDEC2 shows 100% MG in the presence of 150 mM NaCl in the imbibition media but not in 250 mM (MG = 73%; Supplementary Figure S2). UDEC2 germinating seeds have been previously described not only as moderate salt tolerant but also osmotic resistant (305 g/L of PEG), even when both types of stresses are combined (200 mM NaCl plus 175 g/L PEG; [45]). Accordingly, the classification of UDEC2 as moderate salt stress tolerant was confirmed, whereas UDEC4 could be described as high tolerant. However, neither UDEC2 nor UDEC4 seeds germinate at 500 mM NaCl in the imbibition medium (data not shown). Considering these results, 250 mM NaCl concentration was selected for subsequent analyses.

**Figure 2:**
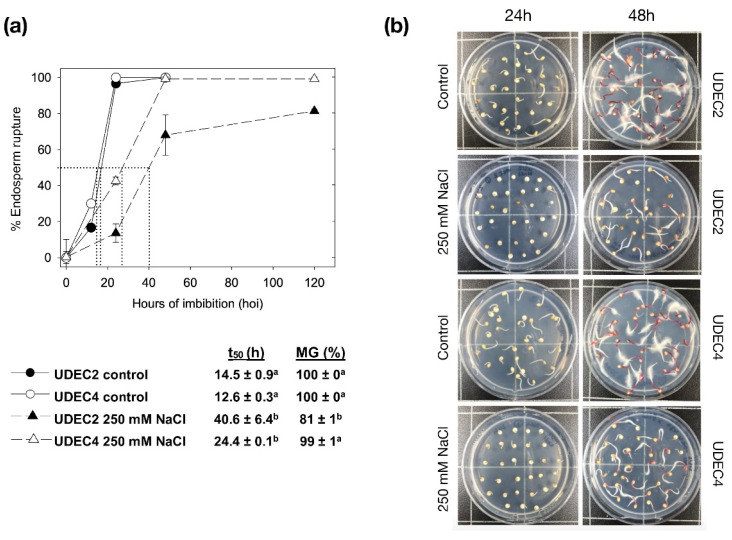
Germination assays of *C. quinoa* one-year-old seeds. (**a**) Germination of UDEC2 and UDEC4 quinoa accessions in control (MS/2 medium) and in the presence of 250 mM NaCl. Time to obtain the 50% of germination (t_50_) and maximum germination percentage (MG %) are indicated. (**b**) Images of UDEC2 and UDEC4 accessions at 24 and 48 h of seed imbibition in control and 250 mM NaCl. Data are means ± standard error (SE) of three technical replicates. Statistically significant differences are indicated with different letters (*p*-value ≤ 0.05).

Further germination assays were conducted using one-year-old seeds from UDEC2 and UDEC4 accessions, both in 250 mM NaCl and under control conditions. In Figure 2a, these seeds from UDEC2 and UDEC4 germinate significantly slower at 250 mM NaCl (t_50_ = 40.6 ± 6.4 h and t_50_ = 24.4 ± 0.1 h, respectively) than the control (t_50_ = 14.5 ± 0.9 h and t_50_ = 12.6 ± 0.3 h, respectively). Moreover, UDEC2 seeds achieve 81% MG, while UDEC4 attains 99% MG. The establishment of seedlings in both accessions proceeded without any significant issues when 250 mM NaCl was added to the imbibition medium (Supplementary Figure S2). 

Notably, two-year-old UDEC2 and UDEC4 seeds show higher t_50_ values than those for one-year-old seeds, both in control and salt stress conditions (250 mM NaCl; Figure 2a and Supplementary Figure S2). This difference in seed germination behavior is most probably caused by aging, a complex trait for orthodox and recalcitrant seeds. Seed aging is a major factor controlling seed vigor where chemical reactions can damage proteins, lipids, and nucleic acids, and eventually impair germination [46]. FAO has pointed out the necessity of the creation of Genebanks with more specific protocols for the conservation of species (https://www.fao.org; accessed on 15 August 2023). Currently, this aspect requires further investigation in quinoa to determine the proper grain storage conditions. This information will be useful not only for seed biology research but also for farmers and the seed industry.

### 2.2. Selection of Candidate Reference Genes

Gene expression analysis is a major issue for studying how organisms respond to environmental conditions [47]. In this context, the use of qPCR and the accuracy of its results depends on proper normalization, often achieved by selecting appropriate reference genes. The ideal reference gene should maintain stable expression across various cell types, tissues, time points, and experimental conditions [22]. An extensive literature review of expression studies published in high-impact journals during 1999 revealed that in over 90% of cases, the *GAPDH*, *ACTB*, *18S*, and *28S-rRNA* housekeeping genes were used for normalization [48]. Numerous studies reported that housekeeping gene expression can vary considerably, hampering the validity of the conclusions [19]. Therefore, rigorous validation of candidate reference genes is crucial to ensure the accuracy of gene expression analysis [49]. 

Upon seed imbibition, dramatic changes in the transcriptional profiles occur from early germination [21,50]. Even more, endosperm and embryo tissues show different transcriptional profiles due to their distinct functions during seed dormancy and germination [23]. Some of the traditional reference genes used in vegetative tissues are not suitable for normalizing gene expression in seeds. Thus, there is a need for reference gene identification in the different stages of the seed, especially in those species like quinoa, where they have not been described yet.

In this study, six reference genes were methodically chosen, derived from previous research on *Chenopodium quinoa* and on seeds of other plant species. Among the selected reference genes are *Glyceraldehyde-3-phosphate dehydrogenase* (*GAPDH*), *Monensin sensitivity 1* (*MON1*), and *Polypyrimidine tract-binding protein* (*PTB*) that have previously been reported as reference genes in quinoa [24,41,51,52]. Additionally, *Actin-7* (*ACT7*), *Ubiquitin-conjugating enzyme* (*UBC*), and *18S ribosomal RNA* (*18S*) were included in the selection due to their established use as reference genes in seeds of other plant species, such as *Arabidopsis thaliana*, *Chenopodium album*, *Lepidium sativum*, and *Sisymbrium officinale* [17,22,35]. *EF1α* (AUR62020767) has been previously reported as a reference gene in quinoa seedlings [41,52]. However, a search in the *Phytozome* database (https://phytozome-next.jgi.doe.gov/; accessed 15 November 2021) [53] using the *EF1α* gene sequence revealed the existence of 12 homologous genes with high similarity (>85%); thus, *EF1α* was excluded from the candidate gene list.

Data of the selected genes used in this study are summarized in Table 1, including reference gene ID, primer sequences, and the biochemical characteristics of the primers. The specificity of the primer combinations was assessed through both melting curve analysis and electrophoretic separation of the PCR product (Table 1 and Supplementary Figures S3 and S4). This evaluation confirmed the specificity of the primer pairs, as indicated by the presence of a single reaction product of the expected size, as shown in Supplementary Figure S4. The qPCR melting curve analysis consistently exhibits a unique peak (Supplementary Figure S3). Amplification efficiencies of the different primer combinations range from 87.0% to 95.1%, while corresponding regression coefficients (r^2^) vary from 0.91 to 0.99 (Table 1 and Supplementary Figure S5), considered as suitable values for the subsequent stability analysis.

### 2.3. Stability of Candidate Reference Genes during Seed Germination under 250 mM NaCl in UDEC2 and UDEC4 Accessions

The expression levels of the candidate reference genes have been quantified through qPCR, using raw cycle threshold (Ct) values. Ct is the cycle in the exponential phase where a significant fluorescence signal is reached when exceeds background levels [54]. 

**Figure 3:**
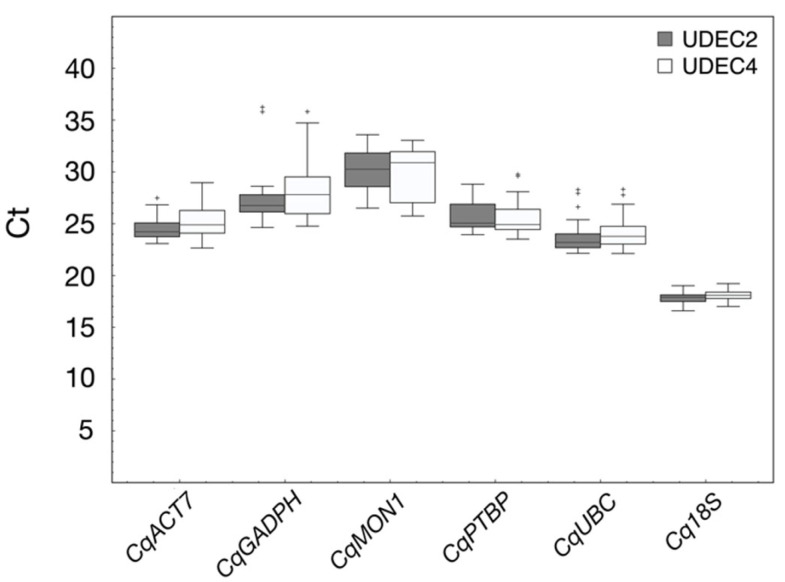
Ct variation of the selected candidate reference genes in UDEC2 and UDEC4 accessions. Values of three biological replicates of 0, 24, 48, 72, and 120 hoi in absence and presence of 250 mM NaCl (n = 48). Boxes represent the 1/4 and 3/4 quartile values. Median is represented as a line across the box, whiskers represent maximum and minimum values, and + symbols are outliers.

In this work, the expression stability of *CqACT7*, *CqGAPDH*, *CqMON1*, *CqPTB*, *CqUBC*, and *Cq18S* genes during seed germination has been evaluated in UDEC2 and UDEC4 accessions under control and salt stress conditions (250 mM NaCl). Samples were taken at the dry seed stage and at several time points of seed germination: 12, 24, and 48 hoi in the control (0 mM NaCl) and at 24, 48, 72, and 120 hoi in the presence of 250 mM NaCl in the imbibition medium. RNA purified from the samples was checked for purity and integrity using spectrophotometry and electrophoresis, respectively (Supplementary Figure S6; Supplementary Table S1). The six reference genes selected display a wide expression range, with Ct values between 16.59 and 36.24 (Figure 3), distributed into different expression level categories: high (*Cq18S*), moderate (*CqACT7* and *CqUBC*), and low abundance (*CqGAPDH*, *CqMON1*, and *CqPTB*). Ct values <40 are only used for the calculation of the PCR efficiency (Table 1; Supplementary Figure S5). Among the six genes, *CqACT7*, *CqUBC*, and *Cq18S* show the lowest variation in expression among the 24 samples. In contrast, *CqGAPDH* and *CqMON1* show the highest variation in expression in all samples (Figure 3).

**Figure 4:**
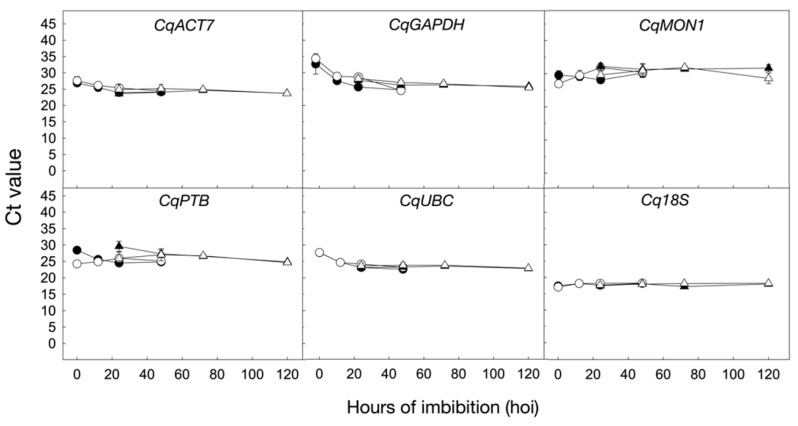
Expression profiles of the selected candidate reference genes upon seed germination in the absence (control: 0, 12, 24, 48 hoi) and presence of 250 mM NaCl (24, 48, 72, 120 hoi). Ct values for UDEC2 samples are represented with dark circles (●) in the control and in the presence of 250 mM NaCl with open circles (○). Ct Values for UDEC4 are denoted with dark triangles (▲) in the control and with open triangles (△) in the presence of 250 mM NaCl. Data are means ± standard error (SE) of two technical replicates of three biological samples.

Figure 4 shows the Ct values of the six candidate reference genes in the 24 samples analyzed. The expression profiles of the *CqACT7*, *CqUBC*, and *Cq18S* candidate genes across the samples in the presence and absence of 250 mM NaCl exhibit no significant differences in both UDEC2 and UDEC4 accessions and remain stable throughout the conditions studied (Figure 4). However, *CqGAPDH*, *CqMON1*, and *CqPTB* expression profiles are less stable as shown graphically, and in accordance with the data shown in Figure 3.

**Figure 5:**
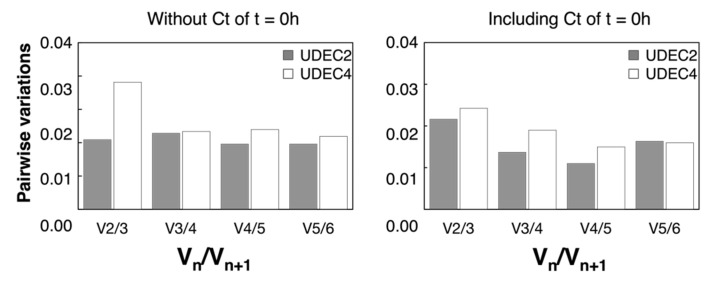
Determination of the optimal number of reference genes. Pairwise variation analysis of selected candidate reference genes in seeds of UDEC2 and UDEC4 *C. quinoa* accessions. Variation value (V_n_) is calculated for a given number (n) of reference genes (according to Section 3.8 of the Materials and Methods section). Ct obtained from samples at dry seed stage are included in the right graph and excluded in the left graph. All pairwise variation values (V_n_/_n + 1_) are below the recommended cut-off of 0.15, indicating that the inclusion of an additional reference gene is not required (n = 48).

Table 2 summarizes the stability of the candidate genes assessed by the *RefFinder* tool that integrates four computational algorithms (*geNorm*, *NormFinder*, *BestKeeper*, and the comparative *ΔCt* method). According to the gene stability rankings derived from the four programs, *RefFinder* assigns an appropriate weight to each gene and calculates the geometric mean of weights for the final rankings. The *geNorm* has been used to calculate the M value, where a smaller M value indicates higher gene stability [55]. Likewise, in *NormFinder*, the SV value of the reference gene according to variance analysis represents the stability. The lower the S value, the more stable they are [56]. In the *BestKeeper* algorithm, the most stable genes show the lowest SD values, and in the *ΔCt* algorithm (based on the *ΔCt* method), genes with lower SD values have more stable expression [57]. The results show that *RefFinder*, *geNorm*, *NormFinder*, and *ΔCt* methods placed *CqACT7* and *CqUBC* as the most stable genes for normalization of expression data during UDEC2 and UDEC4 seed germination in the absence and presence of 250 mM NaCl. Instead, *BestKeeper* placed *Cq18S* and *CqACT7* as the most stable genes (Table 2). Many previous studies reported that a single reference gene was not adequate for normalization. The expression results would generate a 3–6.4-fold error using only one internal reference gene. The optimal number of reference genes to analyze the 24 samples is determined by calculating pairwise variation (V_n_/V_n+1_) by *geNorm*. A value of V_n_/V_n+1_ less than 0.15 indicated that the most suitable reference gene number is n without introducing n + 1 [55]. The appropriate number of genes required for normalization in this study is two, whether including dry seed in the analysis or not (Figure 5).

Data in Figure 4 and Table 2 point out *ACT7*, *UBC*, and *18S* as the most stable reference genes upon seed germination in both species under salt stress. *ACT7*, *UBC*, and *18S* have been widely used as reference genes upon seed germination in several species, such as *Arabidopsis thaliana*, *Brassica rapa*, *Lepidium sativum*, *Sisymbrium officinale*, and *Chenopodium album* [22,30,33,35]. Since transcriptional changes in seeds upon germination usually are specific and different from those in other vegetative plant tissues, it is not unexpected that reference genes previously described in seeds in other species could be suitable for *C. quinoa* upon seed germination [21,22,23]. Additionally, *ACT* has been used as a reference gene to normalize the expression in the halophyte *Suaeda* spp. under salt stress [58]. Similarly, to our results, *GAPDH* is not a suitable reference gene in rice under water-deficient conditions because of the high expression instability [59]. It is noteworthy that the expression analysis of *CqACT7*, *CqGAPDH*, *CqPTB*, and *CqUBC* in the dry seed stage is slightly superior compared to that upon germination. More than 12,000 different types of mRNAs are stored in the dry seed of *Arabidopsis thaliana*, including mRNAs needed for the repair of cellular damage and for initiation of the germination process [21,60]. This specific expression profile in the dry seed could justify the gene expression differences found in our study.

### 2.4. Validation of Selected Reference Genes during Seed Germination by Evaluation of CqMAN7 and CqABI5 Expression

Further validation of candidate genes was conducted by analyzing the expression of *CqMAN7* and *CqABI5*, orthologous genes to *AtMAN7* (*At*5g66460) and *AtABI5* (*At2g36270*) in the model plant *Arabidopsis thaliana* (https://phytozome-next.jgi.doe.gov/; accessed on 3 April 2023). The *AtMAN7* gene not only is highly expressed upon seed germination but also its transcript abundance increments in response to abiotic stress (salt stress; Supplementary Figure S7) [17,61]. The *AtABI5* gene encodes a major bZIP transcription factor involved in ABA signaling during seed maturation and germination and its expression also augments in response to salt stress [26] (Supplementary Figure S7). Considering their expression patterns in *A. thaliana*, both genes were selected for further validation in *C. quinoa* seeds.

**Figure 6:**
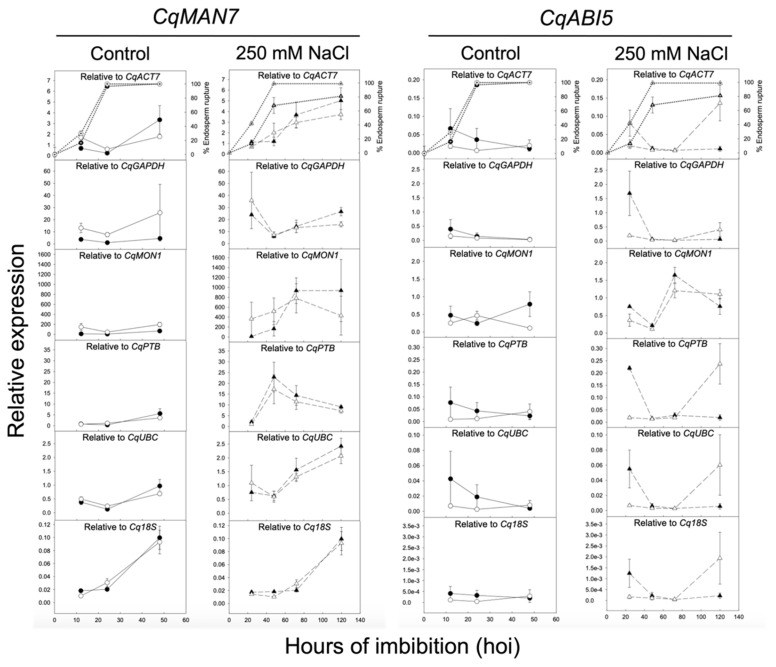
Expression profile of *CqMAN7* and *CqABI5* gene upon seed germination in the absence (control: 12, 24, and 48 hoi, left panel) and presence of 250 mM NaCl (24, 48, 72, and 120 hoi, right panel, dashed lines). *CqMAN7* expression is calculated in each graph using one of the selected reference genes. Expression values for UDEC2 samples are represented with dark circles (●) in the control and with dark triangles (▲) in the presence of 250 mM NaCl. Expression values for UDEC4 are denoted with open circles (○) in the control and with open triangles (△) in the presence of 250 mM NaCl. The seed germination profile is shown in the top graphs (right axis, dotted lines) for UDEC2 (control = dark crossed circles; 250 mM NaCl = dark crossed triangles) and for UDEC4 (control = open crossed circles; 250 mM NaCl = open crossed triangles). Data are means ± standard error (SE) of two technical replicates of three biological samples.

**Figure 7:**
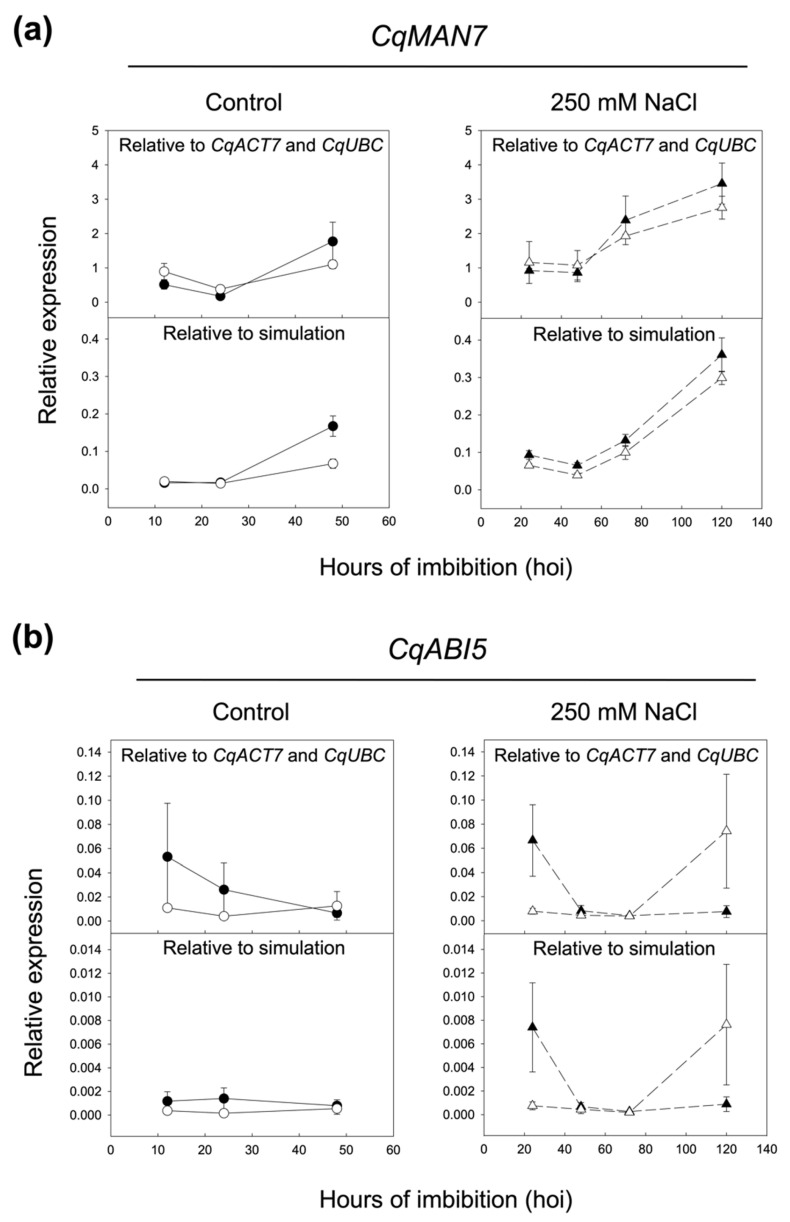
Expression profile of *CqMAN7* and *CqABI5* normalized to *CqACT7* + *CqUBC* and optimal simulated reference genes in UDEC2 and UDEC4 germinating seeds in the absence (control: 12, 24, 48 hoi, left graphs) and presence of 250 mM NaCl (24, 48, 72, 120 hoi, right panel, dashed lines). (**a**) Expression of *CqMAN7* and (**b**) expression of *CqABI5*, relative to the geometric mean values of the two genes selected as most stable reference genes (*CqACT7* and *CqUBC*) and to a theoretical optimal reference gene with a constant Ct value of 20 (simulation). Expression values for UDEC2 samples are represented with dark circles (●) in the control and with dark triangles (▲) in the presence of 250 mM NaCl. Expression values for UDEC4 are denoted with open circles (○) in the control and with open triangles (△) in the presence of 250 mM NaCl. Data are means ± standard error (SE) of two technical replicates of three biological samples.

The expression of *CqMAN7* and *CqABI5* genes were analyzed upon seed imbibition at 12, 24, and 48 hoi in control conditions (0 mM NaCl) and at 24, 48, 72, and 120 hoi in stress conditions (250 mM NaCl) in UDEC2 and UDEC4 accessions. The *CqMAN7* and *CqABI5* expression data were first normalized using each of the candidate reference genes individually (Figure 6). Since *CqACT7* and *CqUBC* were ranked as the most stable candidate reference genes, normalization using both genes was also performed (Figure 7; Table 2). Additionally, the expression data of *CqMAN7* and *CqABI5* genes were normalized with a fixed Ct value of 20 to simulate expected expression patterns (Figure 7). Subsequently, potential disparities between the simulated and the expression profiles of *CqMAN7* and *CqABI5* genes, including differences in the number and positions of expression peaks, were evaluated.

The data of the expression analysis indicate that when normalizing the Ct values of *CqMAN7* and *CqABI5* against the reference genes *CqGAPDH*, *CqMON1*, and *CqPTB*, the resulting expression patterns significantly deviate from the simulated patterns in terms of their shape and peak positions (see Figure 6 and Figure 7). This suggests that *CqGAPDH*, *CqPTB*, and *CqMON1* are not suitable as reference genes for seed germination, both in control conditions and under salt stress in quinoa, being in accordance with the results of Section 2.3. However, when *CqMAN7* and *CqABI5* expression data are normalized against *CqACT7*, *CqUBC*, and *Cq18S*, the obtained expression patterns closely match the expected patterns predicted by the simulation. Moreover, the combination of the two best-performing reference genes for normalization in both accessions (determined by *RefFinder* as *CqACT7* + *CqUBC*; see Figure 7a,b) shows expression patterns that closely resemble the simulated patterns. The *CqMAN7* expression pattern normalized to *CqACT7*, *CqUBC*, or both genes together exhibits a progressive increment of transcripts as germination progresses, reaching a peak at maximum germination in both accessions (UDEC2 and UDEC4), both in the absence and presence of NaCl (Figure 6). Similar expression profiles upon seed germination have been described for *MAN*7 ortholog genes in *A. thaliana*, *Brachypodium distachyon*, *Brassica rapa*, *Hordeum vulgare*, *Lepidium sativum*, *Sisymbrium officinale*, and *Solanum lycopersicum*. Germination *sensu stricto* is caused by two antagonistic forces: the increment of the embryo growth potential and the decrease in the resistance of the seed-covering layers. The *MAN7* expression has been associated with the softening of the embryo covering layers during germination *sensu stricto*, facilitating the radicle emergence [17,18,33,34,62].

*ABI5*, a bZIP transcription factor, is mainly present in seeds and is related to the inhibition of germination and post-germinative growth under unfavorable conditions. ABA induces ABI5 to repress seed germination in response to an eventual abiotic stress [63]. The expression pattern of *CqABI5* in control conditions is almost null in both UDEC2 and UDEC4 accessions. When quinoa seeds undergo salt stress, *CqABI5* transcripts increment at early imbibition in UDEC2 and post-germination stage in UDEC4. High levels of *ABI5* transcripts accumulate in seeds but sharply decline during seed germination. The *CqABI5* expression profiles are in accordance with those published in Arabidopsis [64]. Additionally, the analysis of *CqABI5* expression in dry seeds, when normalized against each of the six reference genes, indicates a decline in expression during early imbibition, consistent with observations in Arabidopsis (see Supplementary Figure S8). 

## 3. Materials and Methods

### 3.1. Plant Materials and Growth Conditions

The first population of UDEC2 and UDEC4 *Chenopodium quinoa* accessions was grown under greenhouse conditions (25 °C/18 °C day/night temperature and about 80% relative humidity; two-year-old seeds). UDEC2 and UDEC4 genotypes, originating from Chile, produce ochre and yellow grains (hereafter seeds) with an average seed weight of 2.8 mg and 3.8 mg (Supplementary Figure S1) [25]. These seeds, which were two years old, were used for an initial germination assay (Supplementary Figure S2). Seeds were surface sterilized in 70% ethanol for 2 min, then in a solution containing 5% (*v*/*v*) sodium hypochlorite, 5% (*w*/*v*), Sodium Dodecyl Sulfate (SDS) for 12 min, and finally rinsed five times with sterile water. Seeds were sown in Petri dishes with half-strength basal Murashige and Skoog (MS/2; Duchefa Biochemie, Haarlem, The Netherlands) medium supplemented with 0.1% sucrose at 22 °C ± 1 °C, >60% relative humidity, and under a long-day condition photoperiod (16 h/8 h; light/darkness) in a germination chamber with a light intensity of 180.4 μmol/m^2^/s. After 3 days, seedlings were transferred individually to soil: vermiculite (3:1) 25 cm diameter pots to the greenhouse (map coordinates: 40.40, −3.83 DD) under the same photoperiod and 18 °C/23 °C (day/night) temperature conditions. Greenhouse lighting was a combination of natural plus artificial light based on high-pressure sodium lamps. The maximum and minimum greenhouse temperatures were maintained by evaporative cooling and gas heating systems, respectively. After six months, seeds were harvested and stored at 21 °C and 30% relative humidity until used for germination assays and for RNA isolation (one-year-old seeds) (Figure 2a).

### 3.2. Light Microscopy: Fixation, Embedding, Sectioning, and Polysaccharide and Protein Histological Staining

For the histological analysis, seeds were sterilized and germinated as indicated in the above section. Imbibed seeds were collected and infiltrated with the FAE solution (Formaldehyde: Acetic acid: Ethanol: water, 3.7:5:50:41.3 by volume) for 20 min under vacuum (33 mbar) at 4 °C (3x), and then incubated at room temperature for 48 h with gentle shaking. Subsequently, seeds were rinsed twice with Phosphate Buffer Sodium solution (PBS) 1× for 30 min while shaking. A graded series of aqueous ethanol mixtures (30%, 50%, 70%, 95%, 100%, 100%) were used for at least 8 h to dehydrate the seeds. The ethanol was then gradually replaced with HistoClear II^®^ (50%, 100%; National Diagnostics; Atlanta, GA, USA) and later embedded in paraffin [33]. Thin sections of 10 μm were performed using a Leica HistoCore Nanocut R microtome (Leica Biosystems, Wetzlar, Germany), collected on glass slides, and dried at 42 °C. Finally, sections were de-waxed in HistoClear II^®^ (10 min, 2×) and gradually hydrated by sinking for 10 min in series of aqueous ethanol mixtures (100%, 95%, 70%, 50%, sterile H_2_O). 

The sections were firstly stained with 0.05% toluidine blue for 30 s and rinsed with sterile water for checking tissue integrity (data not shown). Then, samples were stained with 0.5% (*w*/*v*) periodic acid (Merck Millipore, Burlington, MA, USA), Schiff’s Reagent (Merck Millipore), and 1% (*w*/*v*) Naphthol Blue Black (Sigma-Aldrich; St. Louis, MO, USA). After the staining, the sections were mounted in DPX (Merck Millipore) and observed under bright field-confocal microscope Zeiss LSM 880 (Zeiss, Oberkochen, Germany).

### 3.3. Germination Assays

For germination experiments, *C. quinoa* seeds were surface sterilized as described in Section 3.1. Seeds were not stratified at 4 °C for 4 days to avoid influencing their dormancy status. Three technical replicates of 30 seeds were imbibed on half-strength basal Murashige and Skoog medium (MS/2, Duchefa Biochemie) supplemented with 0.1% sucrose and including 0 mM, 150 mM, and 250 mM NaCl (Figure 2 and Supplementary Figure S2). Although assays have been performed at least three times (biological samples), a unique experiment is represented. Plates were incubated in a germination chamber with a light intensity of 180.4 μmol/m^2^/s at 22 °C ± 1 °C, >60% relative humidity, and long-day (16 h/8 h; day/night) photoperiod.

Seeds were scored as germinated when the radicle emerges through the endosperm, and it is visible under a magnifying lens (endosperm rupture; ER) as previously described [17]. Germination tests were performed in three samples using three technical replicates. Germination was scored at different germination time points (0, 12, 24, 48, 72, and 120 h of imbibition, hoi). The time to obtain the 50% of germination (t_50_), the maximum percentage of germination (MG %), and the statistical analysis (Student’s *t*-test) were calculated using the GERMINATOR Microsoft Office Excel 16.9 package [65].

Seeds upon germination were also imaged under a stereomicroscope Leica MZ95 (Leica Biosystems).

### 3.4. Total RNA Isolation from Chenopodium quinoa Seeds and cDNA Synthesis

Seeds were imbibed in MS/2 in the absence and presence of 250 mM NaCl as described above, and collected at different timepoints of imbibition (0, 12, 24, 48, 72, 120 hoi), frozen in liquid N_2_, and kept at −80 °C until use. Samples were grinded using a Mikro-Dismembrator S (Sartorius AG, Göttingen, Germany), and total RNA purified by the phenol/chloroform method followed by LiCl precipitation as described by [66]. Before final precipitation, samples were treated with RNase-free DNaseI (Hoffman-La Roche, Basel, Switzerland). Briefly, the grinded samples were resuspended in the extraction buffer (0.4 M LiCl, 0.2 M Tris, 25 mM EDTA, 1% SDS, pH 8) and mixed (1 volume) in three serial steps with (1) chloroform, (2) phenol, and (3) chloroform, keeping the aqueous upper phase in each step. Then, nucleic acids were precipitated in 4 M LiCl at 4 °C (overnight), subsequently resuspended in sterile Milli-Q water, and treated with RNase-free DNase I (Hoffman-La Roche) for 90 min at 37 °C. Residual carbohydrates were precipitated in 40 mM CH_3_COONa (pH 5.2) and 100% ethanol (0.5 volume) at room temperature. Finally, total RNA was precipitated in 300 mM CH_3_COONa (pH 5.2) and 100% ethanol (1 volume) overnight (−20 °C) and then resuspended in sterile Milli-Q water.

Quantity of total RNA was estimated spectrophotometrically by the A_260_ absorbance, and the RNA purity was determined by the 260/280 nm and 260/280 nm absorbance ratios (Supplementary Table S1) by using a NanoDrop One Spectrophotometer (Thermo Fisher Scientific; Waltham, MA, USA). RNA integrity and the lack of DNA contamination was also verified by gel electrophoresis (1% agarose; Supplementary Figure S6).

The complementary DNA (cDNA) was synthesized from 2 μg total RNA using the RevertAid First Strand cDNA Synthesis Kit (Thermo Fisher Scientific) following the manufacturer’s instructions. For each sample, 2 μg of total RNA and 1 μL Oligo (dT)_18_ were mixed with sterile water up to a volume of 12 μL and incubated for 5 min at 65 °C. Then, 4 μL Reaction buffer, 1 μL *Ribolock RNase Inhibitor* (20 U/μL), 2 μL of 10 mM dNTP mix, and 1 μL *RevertAid M-MuLV RT* (200 U/μL) were added (V_F_ = 20 μL). Samples were subjected to 42 °C for 60 min, and subsequently to 70 °C for 5 min to stop the reaction. cDNA samples were stored at −20 °C until use.

### 3.5. Selection of Candidate Reference and Target Genes

Six reference genes have been selected considering previous work in *Chenopodium quinoa* and/or seeds. Genes encoding for Glyceraldehyde-3-phosphate dehydrogenase (GAPDH), Monensin sensitivity 1 (MON1), and Polypyrimidine tract-binding protein (PTB) have been previously used as reference genes in quinoa at different physiological stages and processes [24,41,51,52]. *Actin-7* (*ACT7*), *Ubiquitin-conjugating enzyme* (*UBC21*), and *18S ribosomal RNA* (*18S-RNA*) were selected because of their use as reference genes in seeds in other species, such as *Arabidopsis thaliana*, *Chenopodium album*, *Lepidium sativum*, and *Sisymbrium officinale* [22,30,33,35]. Phytozome v13.0 database [53] (https://phytozome-next.jgi.doe.gov/; accessed on 25 May 2023) was used to search for homologous genes for *ACT7*, *UBC21*, and *18S-RNA* in *Chenopodium quinoa*. The Bio-Analytic Resource for Plant Biology (https://www.BAR.utoronto.ca; accessed on 6 June 2023) was utilized to check gene expression upon seed germination and abiotic stress, as previous selection parameters for reducing initial reference gene list [67]. To validate the robustness of the top-ranked reference genes and to address the inconclusive outcomes produced by the algorithms, we conducted a normalization procedure on the expression data of *MAN7* and *ABI5* genes, known for their involvement in seed germination and abiotic stress response, respectively [17,26]. 

### 3.6. Primer Design

Phytozome v13.0 database was used to search for homologous genes of the selected reference and target genes in *C. quinoa* v1.0 genome. Primer sequences for *CqGAPDH* were taken from [24], and the other selected genes were designed using *Primer3 tool* [68] (https://www.bioinformatics.nl/cgi-bin/primer3plus/primer3plus.cgi; accessed on 25 May 2023). To verify primer specificity, *Phytozome Blast search tool* was used against *C. quinoa* genome and primer and gene sequences aligned using *Clustal Omega tool* [69]. The following primer quality parameters were considered: length between 20 and 24 bp, similar melting temperature (Tm) (maximum 2 °C difference) in a range between 55 and 65 °C, amplicon length between 100 and 150 bp, GC content near to 60%, and lack of self-annealing and primer-dimmer formation.

### 3.7. Quantitative PCR (qPCR)

The qPCR was performed in a LightCycler^®^ 480 v1.5 PCR System (Hoffman-La Roche). For each 20 µL reaction, 4 μL of cDNA (5 times diluted) was mixed with 10 µL SYBR Green Master Mix I (Hoffman-La Roche), 3 μL of each primer (150 nM final concentration), and sterile water up to final volume. The PCR thermal-cycling conditions were set as follows: 95 °C for 10 min for denaturation, 45 cycles of 15 s at 95 °C, 30 s at 60 °C, and 20 s at 7 °C for annealing and extension. The dissociation temperature for each amplicon was calculated by increasing temperature from 60 °C to 97 °C. All analyses were performed in three biological samples and two technical replicates. Each amplicon was analyzed by electrophoresis for size confirmation (Supplementary Figure S4). 

Quantitative PCR efficiency was estimated via a calibration dilution curve and slope calculation. The cDNA template was diluted into four gradients (1, 10^−1^, 10^−2^, and 10^−3^), and qPCR detection performed with these serial gradient concentrations of the cDNA template to generate standard curves for estimation of the correlation coefficient (R2) and the amplification efficiency. The correlation coefficient (r^2^) is given for the regression line of the log of the starting quantity (dilution determined; x axis) and the corresponding Ct value (y axis). The following formula E = (10^−1/slope^ − 1) × 100 is used to calculate the PCR amplification efficiency of each target gene and reference gene (Supplementary Figure S5). We used LightCycler480 Software (Hoffman-La Roche).

Expression levels were determined as the number of cycles needed for the amplification to reach a threshold fixed in the exponential phase of the PCR (C_t_) [54]. To compare data from different cDNA samples, expression values for *CqMAN7* and *CqABI5* genes were normalized with the corresponding expression values to the selected reference genes [17,24]. Additionally, the anticipated expression patterns for *CqMAN7* and *CqABI5* were simulated. During this simulation, the Ct values of the target genes were normalized against a constant Ct value of 20, which serves as a theoretical optimal reference gene. This entailed replacing the Ct value of the prospective reference genes with a uniform value of 20 across all sampling points and biological replicates, solely for simulation purposes. Consequently, we performed a comparative analysis between the simulation and the resultant expression profiles of the target genes. A reference gene is deemed unsuitable if the normalization outcomes significantly deviated from the simulated values. 

### 3.8. Gene Stability Analysis

Reference gene stability was analyzed by *geNorm* [55], *Normfinder* [56], *Bestkeeper* [70], and the comparative Delta C_t_ (−ΔC_t_) [57] methods using RefFinder tool [71]. Standard deviation of the C_t_ values obtained for each *C. quinoa* line is calculated for Delta C_t_ and Bestkeeper methods and the stability value (SV) and M-value (MV) for *Normfinder* and *geNorm*, respectively. Selected reference genes were ranked according to their stability in each method and an overall comprehensive ranking integrating all four methods is also obtained by *RefFinder*.

The minimum number of reference genes required for normalization of target gene expression was determined by the calculation of the pairwise variation of a given number of reference genes. Variation value (V_n_) was calculated until the addition of an extra reference gene had no significant effect, according to the formulas:

NF_n_ = for each sample, geometric mean of the Ct obtained for n reference genes

A_n/n+1_ = for each sample, log_2_ of NF_n_/NF_n+1_ ratio

V_n/n+1_ = standard deviation of A_n/n+1_ dataset

where n (2 ≤ n ≤ 6) stands for the number of reference genes used in the formula. The calculations were made starting with all selected reference genes (n = 6) and the reference gene that ranked the last in *geNorm* method was discarded and the calculation was repeated for the remaining genes until two genes were left.

## 4. Conclusions

In conclusion, this study proposes a model for germination *sensu stricto* in *Chenopodium quinoa* seeds where the breakage of the pericarp and the testa is followed by endosperm rupture (ER), which has been established as the criterion for scoring seed germination. 

The data presented about candidate reference genes support the suitability of *CqACT7* and *CqUBC* as reference genes for normalizing gene expression. The *Refinder* tool ranks these two genes as the most stable out of the six candidates analyzed during seed germination in response to salt stress. The expression profiles of *CqMAN7* and *CqABI5* genes have been used for subsequent validation, and they are in accordance with those reported for orthologous genes in other species. In summary, this study is the first to systematically explore and propose reference genes for normalizing qPCR expression data in *Chenopodiumm quinoa* during seed germination under standard laboratory control and salt stress conditions. These recommended reference genes can be valuable tools in the future for qPCR studies in quinoa seeds.

## Figures and Tables

**Table 1:** List of primers used. Gene ID, primer sequences, dissociation temperature, amplification product size, amplification efficiency, and regression coefficient (r^2^). *CqGAPDH* primer sequences were taken from [24].

Gene ID	Gene Description	Gene Abbreviation	Primer Sequence	Tm (°C) of PCR Product	Amplification Product Size (bp)	Amplification Efficiency (%)	Regression Coefficient (r^2^)
AUR62019117	Actin-7	*CqACT7*	TGAACAGGAATCAGAGACAGCC	83.3	148	95.1	0.99
			CAGAAGACTCCATACCGACTAG				
AUR62005566	Glyceraldehyde-3-phosphate dehydrogenase	*CqGAPDH*	CGGCTTCCTTCAACATCATTCCTAGC	83.3	144	89.7	0.99
			GCCTGACAGTGAGATCAACAACCG				
AUR62020295	Monosensin activity 1	*CqMON1*	GGCGATGAACATAAGCTTGC	79.8	100	91.5	0.99
			TTCCTGCCCGAACTAACTTG				
AUR62034430	Polypyrimidine-tract binding protein	*CqPTB*	CGGAGCATGTGAGTTCATGT	80.6	154	92.7	0.91
			CCAACAACAGGCTGAACAAG				
AUR62036615	Ubiquitin-conjugating enzyme	*CqUBC*	TTGATCAAGGGCCCATCAGAAA	82.3	103	90.3	0.99
			AAAATCGCACTTGAGGAGGTTG				
Scaffold_4105:20989..22796	18S ribosomal RNA	*Cq18S*	GATGTTACTTTTAGGACGCCGC	82.32	108	87	0.99
			TGCCCTTCCGTCAATTCCTTTA				
AUR62007598	Mannan endo-1,4-beta-mannosidase 7	*CqMAN7*	GGAATAGAGTCATATGGAGATGGT	81.2	165	85.5	0.99
			TCCATTCCTTATCTCCCTTGCC				
AUR62028537	Abscisic acid-insensitive 5	*CqABI5*	TTCACCAGCAATGACAGACCAT	82.5	108	106.5	0.99
			TAGATTGAAGACTGGCGCCCC				

**Table 2:** Expression stability of selected reference genes in *C. quinoa* UDEC2 and UDEC4 seeds. Genes are ranked from the most (1) to the least (6) stable gene according to stability values obtained in *geNorm* (MV, M value), *NormFinder* (SV, stability value), *BestKeeper* (SD, standard deviation), and *ΔCq* (SD, standard deviation) methods. *RefFinder* comprehensive ranking (GM, geometric mean) including stability values obtained in all four methods is also shown (n = 48).

**UDEC2**	**Gene**	**RefFinder**		**geNorm**		**NormFinder**		**BestKeeper**		**ΔCt**	
		**Rank**	**GM**	**Rank**	**MV**	**Rank**	**SV**	**Rank**	**SD**	**Rank**	**SD**
	***CqACT7***	1	1.19	1	0.50	1	0.50	2	0.65	1	1.13
	***CqUBC***	2	1.87	1	0.50	2	0.54	3	0.70	2	1.13
	***Cq18S***	3	2.28	2	0.75	3	0.68	1	0.53	3	1.26
	***CqGAPDH***	4	4	3	0.89	4	0.96	4	0.80	4	1.38
	***CqPTB***	5	5	4	1.07	5	1.08	5	1.17	5	1.48
	***CqMON1***	6	6	5	1.41	6	1.95	6	1.78	6	2.10
**UDEC4**	**Gene**	**RefFinder**		**geNorm**		**NormFinder**		**BestKeeper**		**ΔCt**	
		**Rank**	**GM**	**Rank**	**MV**	**Rank**	**SV**	**Rank**	**SD**	**Rank**	**SD**
	***CqACT7***	1	1.32	1	0.68	1	0.18	3	1.08	1	1.32
	***CqUBC***	2	1.68	1	0.68	2	0.51	2	0.76	2	1.35
	***Cq18S***	3	2.63	2	1.07	4	1.36	1	0.51	4	1.78
	***CqPTB***	4	3.66	4	1.45	3	1.13	4	1.40	3	1.38
	***CqGAPDH***	5	4.73	3	1.29	5	1.51	5	1.43	5	1.86
	***CqMON1***	6	6	5	1.70	6	1.96	6	2.05	6	2.20

## Data Availability

Not applicable.

## Supplementary Materials

The supporting information can be downloaded at: https://www.mdpi.com/article/10.3390/ijms242115878/s1.
