# Supplementary material for: Identification of Reference Genes for Precise Expression Analysis during Germination in Chenopodium quinoa Seeds under Salt Stress
Source: Int J Mol Sci. 2023 Nov 1;24(21):15878. doi: 10.3390/ijms242115878 (PMC10650251; doi:10.3390/ijms242115878)
Supplement: Supplementary file 1 [file ijms-24-15878-s001.zip › ijms-2649455-supplementary.pdf]

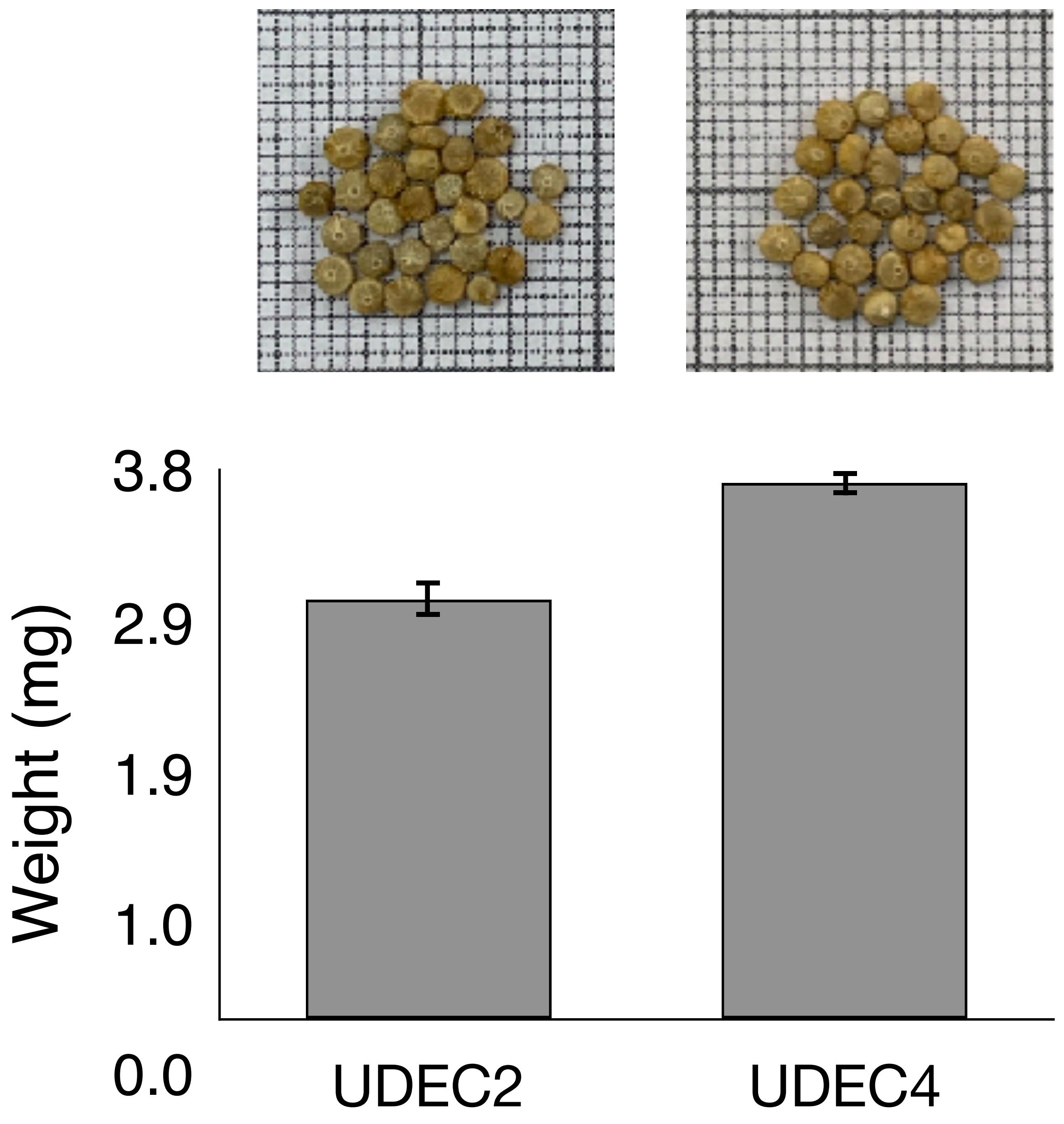

Figure S1. General aspect and average seed weight of UDEC2 and UDEC4 accessions dry seeds (*n*=100).

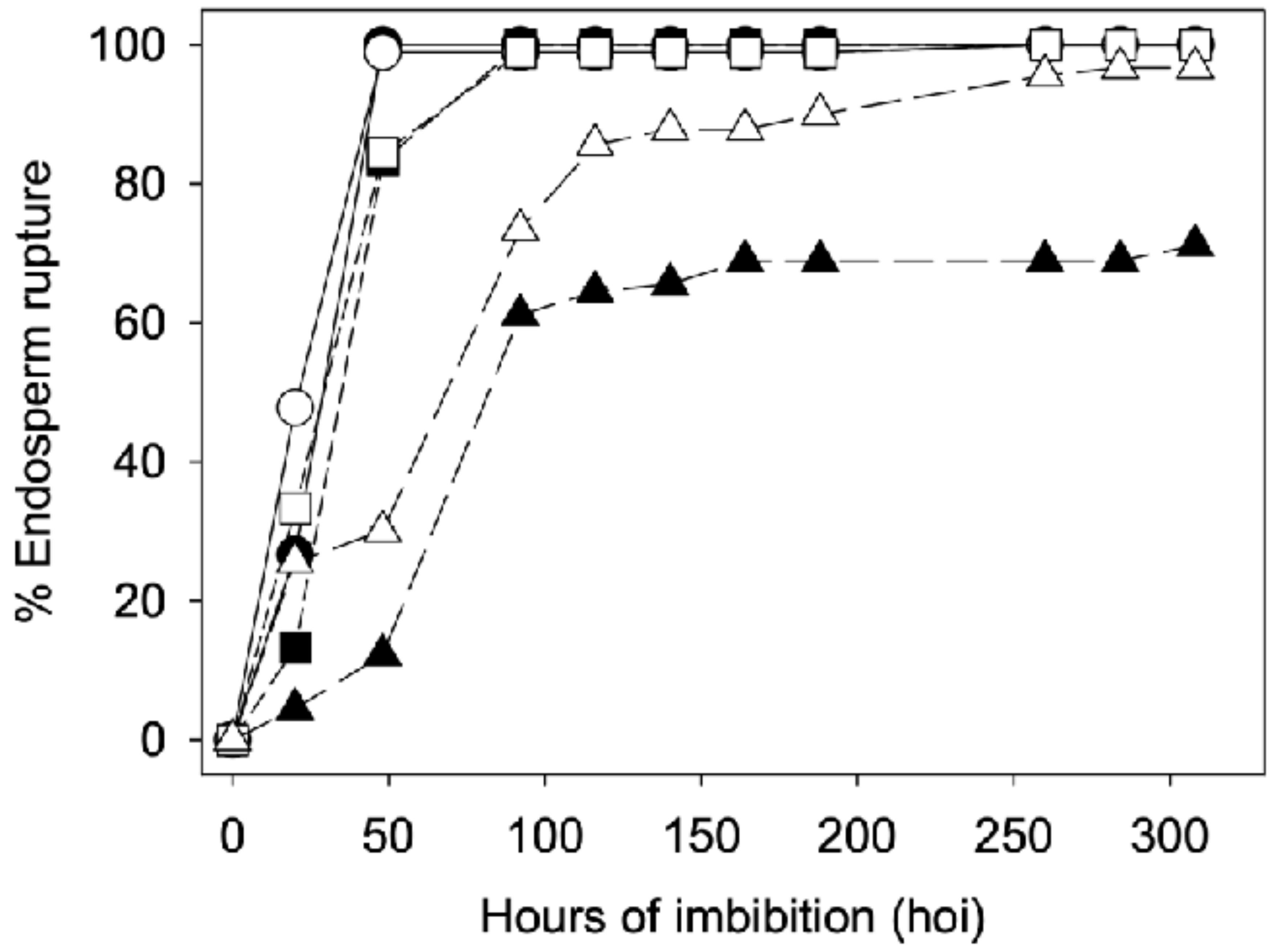

	t_{50} (h)	MG (%)
UDEC2 0 mM NaCl	21.1 ± 0.2[a]	100 ± 0[a]
UDEC4 0 mM NaCl	20.7 ± 0.8[a]	100 ± 0[a]
UDEC2 150 mM NaCl	38.1 ± 5.6[bc]	100 ± 0[a]
UDEC4 150 mM NaCl	43.6 ± 0.5[ab]	100 ± 0[a]
UDEC2 250 mM NaCl	85.5 ± 5.0[d]	73 ± 3[b]
UDEC4 250 mM NaCl	57.0 ± 3.9[c]	98 ± 2[a]

Figure S2. Germination assays of *C. quinoa* two-year-old seeds. Germination profile of UDEC2 and UDEC4 accessions in absence (control), 150 and 250 mM NaCl. Time to get 50% germination (t50) and maximum germination percentage (MG %) are indicated. Percentage of endosperm rupture (ER) is assessed upon germination at 0, 20, 48, 92, 116, 140, 164, 188, 260, 284 and 308 hoi. Data are means ± standard error (SE) of three technical replicates. Statistically significant differences are indicated with different letters (*p*-value ≤ 0.05).

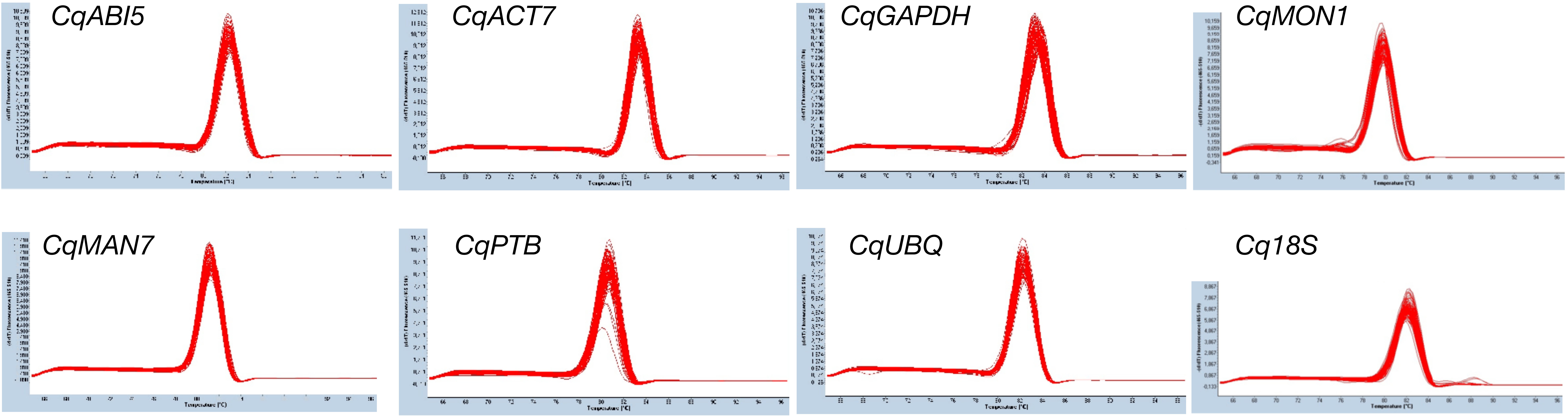

Figure S3. Melting peaks of the candidate reference and target genes.

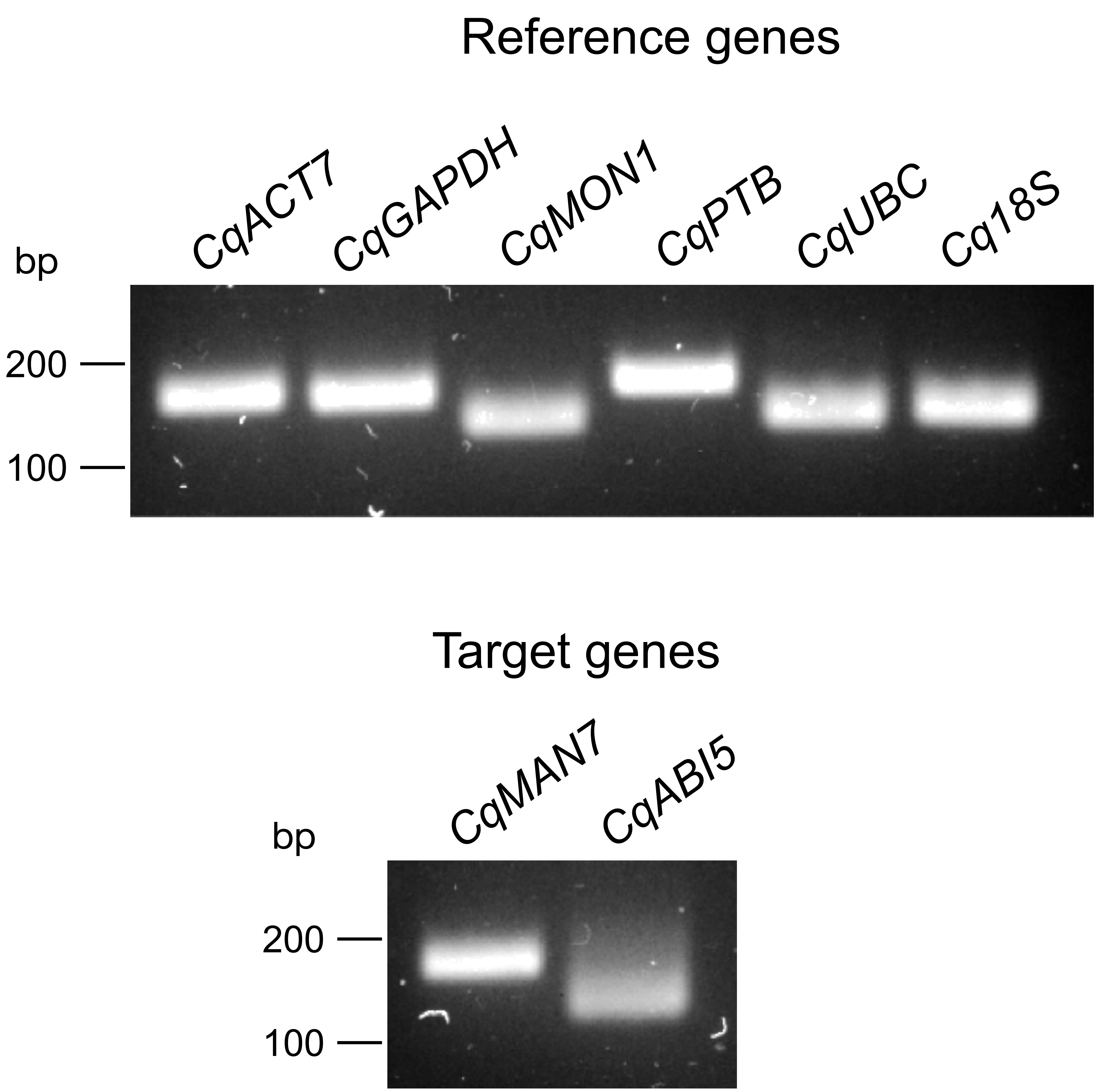

Figure S4. qPCR amplification products of the candidate reference and target genes.

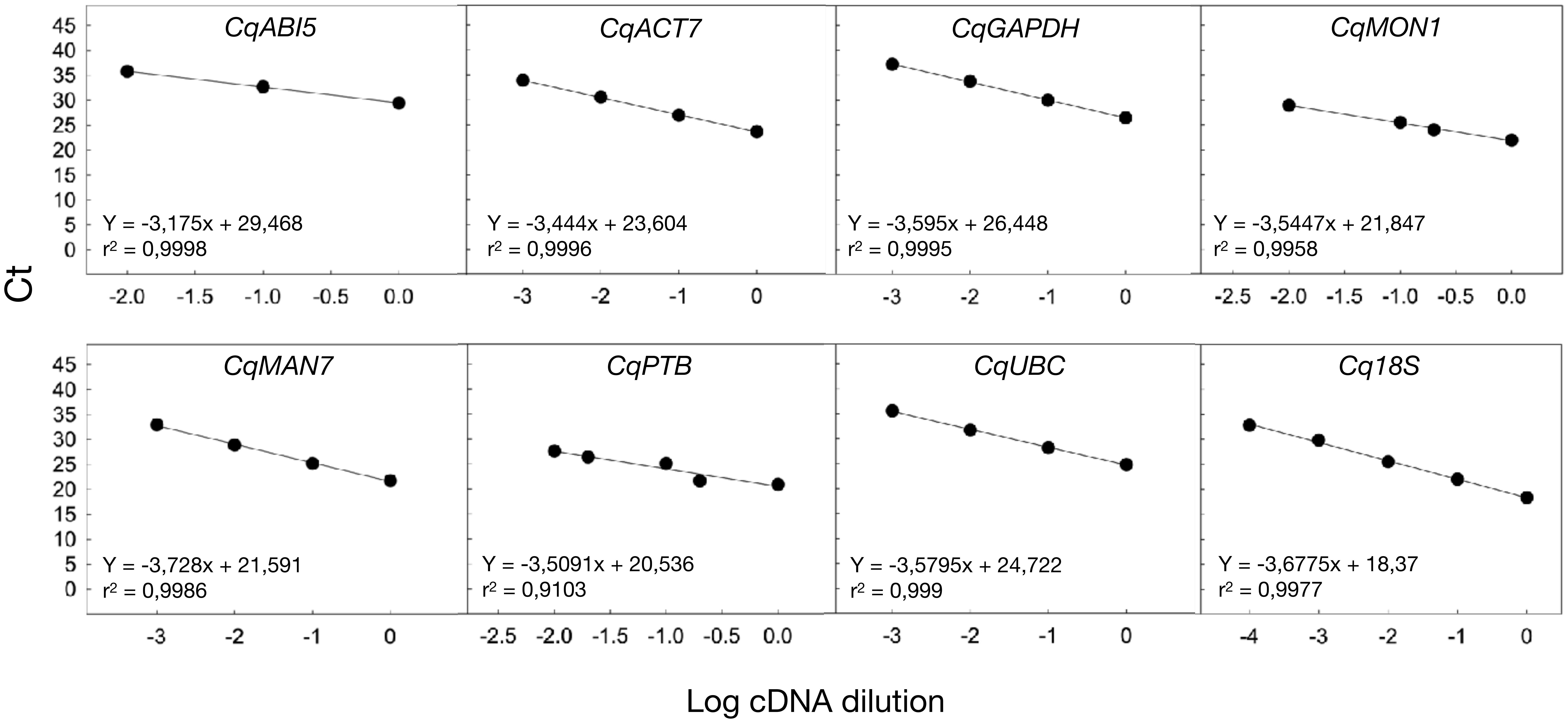

Figure S5. Primer efficiency calculation. Ct obtained for each cDNA dilution is plotted (according to section of 3.7 of materials and methods). The regression line and correlation coefficient (r^2) are shown.

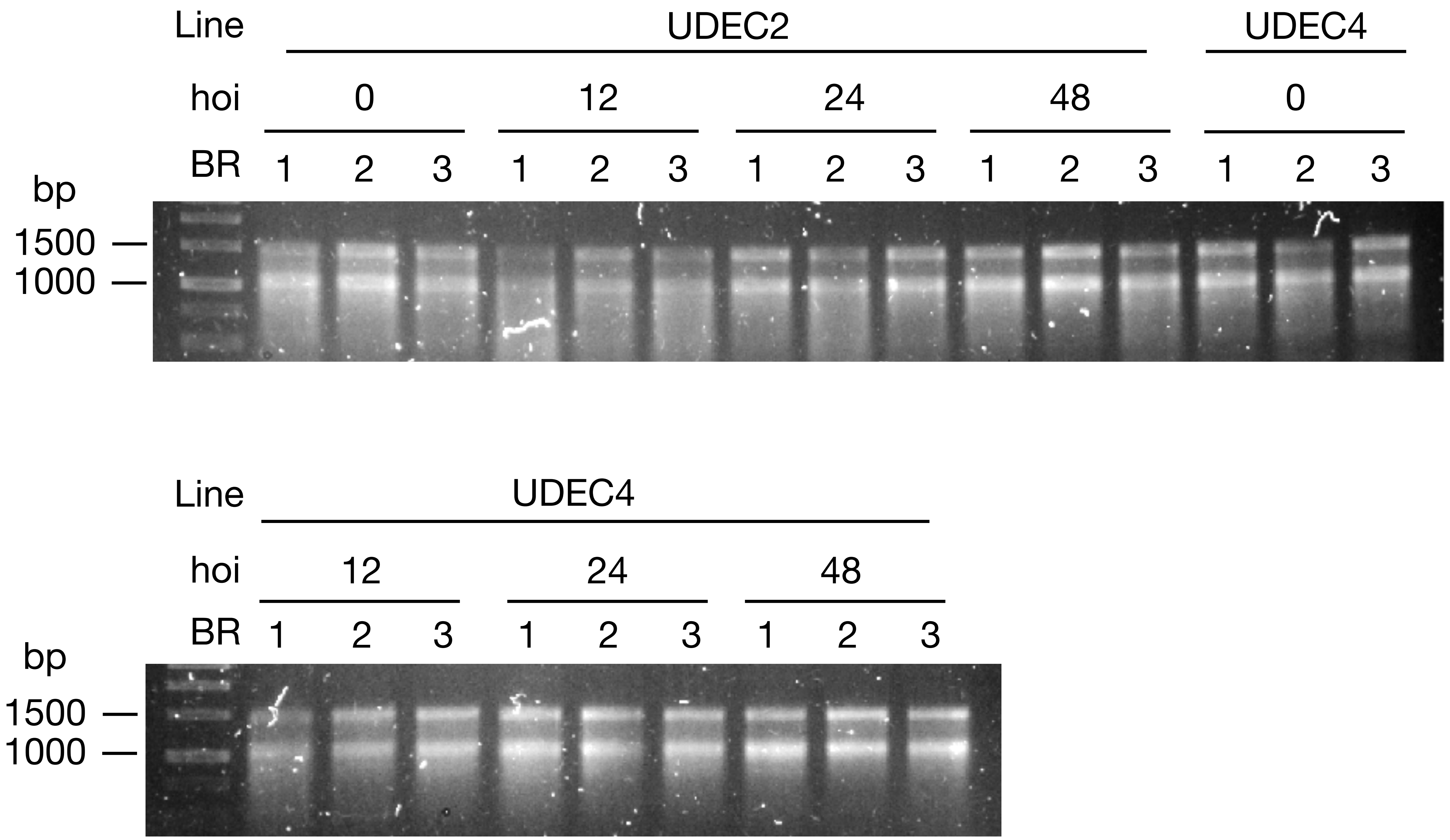

Figure S6. Integrity of total RNA from UDEC2 and UDEC4 seeds. RNA samples (500 ng) are analyzed in 1% agarose gel. DNA molecular weight is indicated in the first lane. Hoi, hours of imbibition; BR, biological replicate; bp, base pairs.

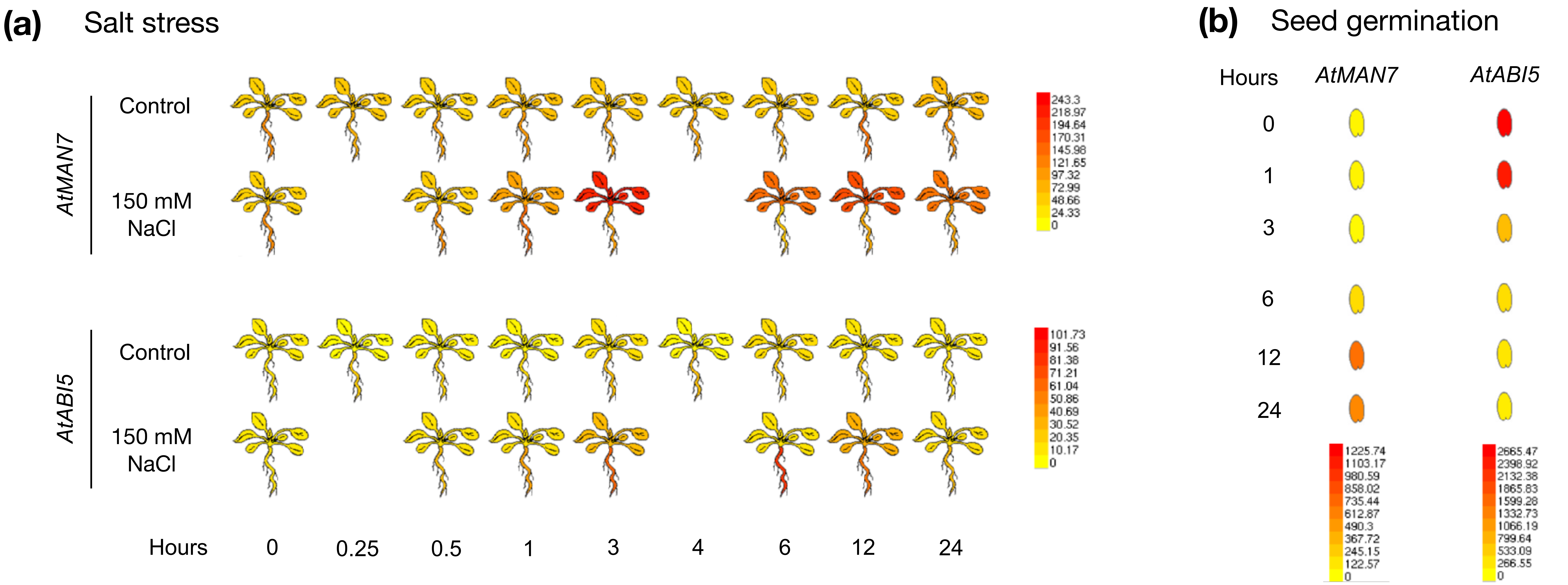

Figure S7. Pictographic representation of *AtMAN7* and *AtABI5* expression levels in *Arabidopsis thaliana*. (a) *A. thaliana* seedlings are coloured according to *AtMAN7* and *AtABI5* expression levels in controls (without salt stress) and in 0, 0.25, 0.5, 1, 3, 4, 6, 12 and 24h after salt stress (150 mM NaCl). (b) Expression levels of *AtMAN7* and *AtABI5* in *A. thaliana* seeds upon 0, 1, 3, 6, 12, 24 hoi. Expression data in *A. thaliana* is taken from the University of Toronto Bio-Analytic Resource for Plant Biology (BAR; https://bar.utoronto.ca/; accessed June 6th 2023).

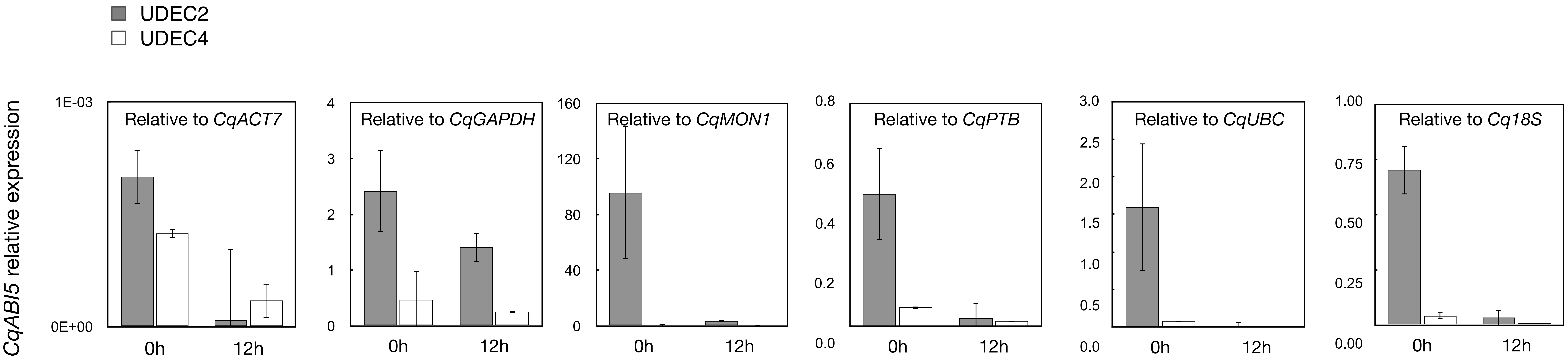

Figure S8. *CqABI5* expression levels at dry seed stage and 12 hoi in UDEC2 and UDEC4 *C. quinoa* seeds. Data are means ± standard error (SE) of two technical replicates of three biological samples.

Supplementary Table S1. Concentration of RNA in samples from UDEC2 and UDEC4 upon seed germination in the absence (control: 0, 12, 24, 48 hoi) and presence of 250 mM NaCl (24, 48, 72, 120 hoi) and their A_{260}/A_{280} and A_{260}/A_{230} ratios.

Quinoa line	Stress	hoi	Biological replicate	Concentration (ng/ul)	Ratio OD260 / 280	Ratio OD 260 / 230
**UDEC2**	control	0	1	2476,3	2,21	2,46
**UDEC2**	control	0	2	998	2,09	2,46
**UDEC2**	control	0	3	2228,3	2,21	2,46
**UDEC2**	control	12	1	2575,4	2,12	2,43
**UDEC2**	control	12	2	2474,4	2,12	2,43
**UDEC2**	control	12	3	2318,6	2,19	2,45
**UDEC2**	control	24	1	1906,7	2,17	2,52
**UDEC2**	control	24	2	3427,4	2,16	2,42
**UDEC2**	control	24	3	1583,2	2,15	2,51
**UDEC2**	control	48	1	980,4	2,12	2,44
**UDEC2**	control	48	2	4389,8	2,19	2,45
**UDEC2**	control	48	3	436,8	2,06	2,47
**UDEC4**	control	0	1	3220,1	2,16	2,45
**UDEC4**	control	0	2	2619,3	2,16	2,4
**UDEC4**	control	0	3	2984,4	2,17	2,44
**UDEC4**	control	12	1	2995,6	2,16	2,44
**UDEC4**	control	12	2	2733,6	2,14	2,3
**UDEC4**	control	12	3	1486,9	2,14	2,51
**UDEC4**	control	24	1	5078,9	2,19	2,42
**UDEC4**	control	24	2	2594	2,13	2,43
**UDEC4**	control	24	3	2458	2,19	2,43
**UDEC4**	control	48	1	6665,1	2,15	2,42
**UDEC4**	control	48	2	5412	2,21	2,45
**UDEC4**	control	48	3	2479,2	2,15	2,49
**UDEC2**	250 mM NaCl	24	1	3220,2	2,17	2,44
**UDEC2**	250 mM NaCl	24	2	2502,2	2,2	2,44
**UDEC2**	250 mM NaCl	24	3	2863	2,15	2,42
**UDEC2**	250 mM NaCl	48	1	4895,9	2,19	2,42
**UDEC2**	250 mM NaCl	48	2	3256,7	2,19	2,45
**UDEC2**	250 mM NaCl	48	3	2235,5	2,18	2,46
**UDEC2**	250 mM NaCl	72	1	3717,5	2,18	2,44
**UDEC2**	250 mM NaCl	72	2	5446,9	2,22	2,42
**UDEC2**	250 mM NaCl	72	3	3818,4	2,19	2,44
**UDEC2**	250 mM NaCl	120	1	4689,9	2,21	2,43
**UDEC2**	250 mM NaCl	120	2	6607,6	2,17	2,41
**UDEC2**	250 mM NaCl	120	3	5637	2,21	2,42
**UDEC4**	250 mM NaCl	24	1	3825,2	2,18	2,43
**UDEC4**	250 mM NaCl	24	2	3702,7	2,17	2,35
**UDEC4**	250 mM NaCl	24	3	3761,2	2,18	2,43
**UDEC4**	250 mM NaCl	48	1	5712,5	2,22	2,42
**UDEC4**	250 mM NaCl	48	2	5179,2	2,21	2,42
**UDEC4**	250 mM NaCl	48	3	4980,4	2,21	2,43
**UDEC4**	250 mM NaCl	72	1	4970,3	2,2	2,41
**UDEC4**	250 mM NaCl	72	2	4321,4	2,2	2,42
**UDEC4**	250 mM NaCl	72	3	5774,6	2,22	2,41
**UDEC4**	250 mM NaCl	120	1	2282,4	2,17	2,51
**UDEC4**	250 mM NaCl	120	2	7011,6	2,17	2,41
**UDEC4**	250 mM NaCl	120	3	5366,3	2,21	2,42
